# Heterochrony as Diachronically Modified Cell-Cell Interactions

**DOI:** 10.3390/biology5010004

**Published:** 2016-01-14

**Authors:** John S. Torday

**Affiliations:** Evolutionary Medicine, University of California-Los Angeles, 621 Young Drive South, Los Angeles, CA 90095-1606, USA; jtorday@ucla.edu; Tel.: +1-310-222-8186; Fax: +1-310-222-3887

**Keywords:** evolution, heterochrony, cell-cell signaling, synchronic, diachronic, growth factor, growth factor receptor

## Abstract

Heterochrony is an enabling concept in evolution theory that metaphorically captures the mechanism of biologic change due to mechanisms of growth and development. The spatio-temporal patterns of morphogenesis are determined by cell-to-cell signaling mediated by specific soluble growth factors and their cognate receptors on nearby cells of different germline origins. Subsequently, down-stream production of second messengers generates patterns of form and function. Environmental upheavals such as Romer’s hypothesized drying up of bodies of water globally caused the vertebrate water-land transition. That transition caused physiologic stress, modifying cell-cell signaling to generate terrestrial adaptations of the skeleton, lung, skin, kidney and brain. These tissue-specific remodeling events occurred as a result of the duplication of the Parathyroid Hormone-related Protein Receptor (PTHrPR) gene, expressed in mesodermal fibroblasts in close proximity to ubiquitously expressed endodermal PTHrP, amplifying this signaling pathway. Examples of how and why PTHrPR amplification affected the ontogeny, phylogeny, physiology and pathophysiology of the lung are used to substantiate and further our understanding through insights to the heterochronic mechanisms of evolution, such as the fish swim bladder evolving into the vertebrate lung, interrelated by such functional homologies as surfactant and mechanotransduction. Instead of the conventional description of this phenomenon, lung evolution can now be understood as adaptive changes in the cellular-molecular signaling mechanisms underlying its ontogeny and phylogeny.

## 1. Introduction

Heterochrony is an enabling concept in evolution theory because it captures the image of dynamic biologic change diachronically across space and time. The term heterochrony (see Table 1 below) was first used by Haeckel in his explanation of the Biogenetic Law [1]. De Beer subsequently used Heterochrony to denote differences in the ontogenies of related taxa [2]. The latter comparative definition is the one in principal use [3]. More recent interest in the question of Heterchrony has arisen because of the integration of developmental biology into evolution theory, or EvoDevo [4]. But this initiative has failed to incorporate the highly mechanistic nature of contemporary Developmental Biology [5] beginning in the late 1970s with the discovery of soluble growth factors and their complementary receptors residing on neighboring cell-types, determining the patterns of morphogenesis [6]. The associations of growth factors with the process of evolution are sometimes described [7], but the significance of the growth factor receptors, down-stream signals and subsequent effects on morphogenesis are never specifically addressed—yet this is a microcosm of what evolution constitutes, if only we would perceive it appropriately [8,9,10]. The only reasonable explanation for such gross oversights is that Cell Biology itself has been overarched by the evolutionists, largely due to an incident of history [11]. Haeckel and Spemann were unable to provide scientific evidence for their theories of the Biogenetic Law, and for the “Organizer”, respectively, so the evolutionists turned to the geneticists to advance their agenda, rejecting the embryologists in the process. As a result, the nominal mechanism of evolution is constituted by genetic mutation and natural selection [12]. If this were indeed the case, then there would be no need to delineate or utilize the principles of cellular-molecular morphogenesis, yet therein lie the fundamental principles of form and function [13]. The following is in service to functionally integrating our contemporary knowledge of the cellular-molecular mechanisms of development with the role of heterochrony in evolutionary biology.

**Table 1:** Background and significance for the reinterpretation of heterochrony.

The concept of heterochrony was first introduced by Ernst Haeckel in 1875 as the mechanistic basis for his Biogenetic Law.
Heterochrony is due to a change in function or form during development.
Kolman (1885) used the term paedomorphosis to describe heterochrony as process for retaining juvenile properties.
De Beer (1930) used the term neoteny as a subcategory of heterochrony to describe the retention of earlier developmental properties.
Peramorphosis is used to describe delayed maturation and extended periods of growth.
In his book “Ontogeny and Phylogeny”, Stephen J. Gould described the significance and importance of heterochrony as the mechanism of evolution. However, he never provided a specific mechanism for how and why such changes occur, obviating the possibility of scientifically testing its hypothesized role in evolution.
Since the late 1970s, the determination of growth and differentiation by soluble growth factor-mediated cell-cell signaling has been acknowledged to be the mechanism of development.
Despite this, the advent of Evolutionary Developmental Biology, or EvoDevo has not assimilated growth factor signaling into its analyses.
The current article demonstrates the value added in understanding heterochrony as a sequence of cell-cell interactions that can be modified by environmental factors to understand how and why evolution has occurred. The power of this approach is in its ability to understand the processes of development, physiology, homeostasis and pathology as one continuous, scale free evolutionary mechanism for the first time.
This explanation of heterochrony offers a change in the language of evolutionary biology, representing what Kuhn [14] referred to as a paradigm shift in his “The Structure of Scientific Revolutions”.

## 2. Normal Embryologic Development, or “Monochrony”, in Contrast to Heterochrony

The spatio-temporal patterns of vertebrate embryogenesis are determined by the elaboration of cell-specific growth factors signaling to their cell surface G-Protein Coupled Receptors are neighboring cells of differing germline origins to form patterns of growth and differentiation, from the zygote [15] to the offspring [16]. This is an iterative process by which the zygote divides, giving rise to the animal and vegetal poles, the blastula, gastrula, and so on [17] during embryogenesis, followed by fetal growth and differentiation [18] to generate the offspring. All of these processes are mediated by growth factor-receptor signaling mechanisms that form the tissues and organs of the body. If the environmental conditions remain unchanged, this process would simply be recapitulated from one life cycle to the next. But the environment is in perpetual flux [19]—climate, topography, seasons, food abundance, competition with other organisms—so organisms must be able to adapt in order to survive using the mechanism we refer to as evolution [20]. This is particularly apparent when environmental conditions are physiologically stressful, since the adaptive changes are both discernable and measurable [21]. Classic examples are the consequences of the five mass extinctions [22], and the transition of plants and animals from water to land [23], brought about by carbon dioxide causing an atmospheric Green House Effect that dried up water sources globally [24]. During that period there were several genetic adaptations that profoundly affected vertebrate physiology, allowing for successful adaptation to terrestrial life [10]. By focusing on those events both ontogenetically and phylogenetically [10], we can envision how heterochrony facilitated vertebrate evolution.

The terrestrial forms of the vertebrate lung, kidney, bone, skin and brain all evolved during the water-land transition in adaptation to terrestrial life. The epitome of the mechanisms underlying these phenotypic changes is the duplication of the Parathyroid Hormone-related Protein Receptor (PTHrPR) [25], expressed in all of these organs by the mesoderm [26] in close proximity to ubiquitous epithelial PTHrP production. The duplication of the PTHrPR gene amplified signal transduction for PTHrP signaling from the endodermal epithelium to the mesodermal fibroblast [27]. In the case of the lung, it facilitated the formation of alveoli [28]; in the kidney, PTHrP signaling amplification generated glomeruli [29]; in bone, increased PTHrP amplification allowed for the five documented phenotypic changes in the skeleton that compensated for the increased effect of gravity on the skeleton relative to buoyancy in water [30]; in the skin, PTHrP fostered barrier formation by skin cells for prevention of water and electrolyte loss [31]; the brain is thought to have evolved from the skin [32], and it has a number of molecular traits that are derivative of the latter at the molecular level [9] that would have facilitated its evolution for land adaptation.

## 3. Lung Evolution as Ontogeny and Phylogeny

In hindsight, it is obvious that land vertebrates had to evolve lungs in order to adapt to air breathing, yet phylogenetically the fish organ of gas exchange—the gill—is an analog of the lung, not the functionally ancestral homolog—counterintuitively, the fish swim bladder, which facilitates buoyancy, is actually the functional homolog of the lung [33]. This example epitomizes the value of a cellular-molecular ontogenetic-phylogenetic approach to evolution. The swim bladder is an adaptation to gravity that utilizes atmospheric gas to inflate or deflate the bladder, aided by the secretion of cholesterol into the air space by the gas gland epithelium. The swim bladder expresses both Cholesterol, the most primitive lung lipid component of surfactant [34], and Surfactant Protein A [35], a host defense peptide that originated phylogenetically from the gut.

The lung has adapted to atmospheric oxygen phylogenetically and ontogenetically by reducing the surface area of the gas exchange unit, increasing the ratio of the gas-exchange surface area to the blood volume for increased oxygenation. Concomitant ontogenetic and phylogenetic increases in the biological activity of lung surfactant secreted into the alveolar space prevented the alveolar collapse that would otherwise have been caused by the increase in surface tension resulting from the decreased surface area (surface tension being inversely related to surface area by the Law of Laplace). The stretch-regulated mechanism of alveolar surfactant production [10] is functionally homologous with the swim bladder, both of which are gravity-sensing mechanisms [30]. By focusing on the developmental/homeostatic cell-cell interactions that have evolved from the gas-exchange unit of fish (swim bladder) to that of land vertebrates to form the alveoli [36], one can see how they were selected for the thinning of the alveolar wall and the anti-atelectatic function of the surfactant system [37].

## 4. The Lipofibroblast as a “Rosetta Stone” for Lung Evolution

**Figure 1:**
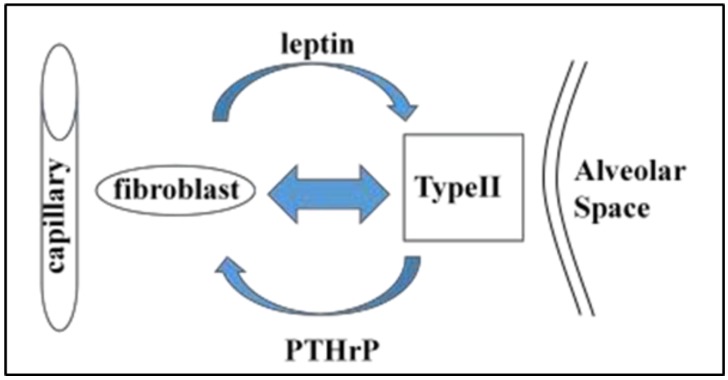
Active recruitment of neutral lipid from lipofibroblasts by alveolar Type II cells. Neutral lipids stored in lipofibroblasts are actively “trafficked” to alveolar Type II cells by means of Adipocyte Differentiation Related Protein (ADRP), regulated by Parathyroid Hormone-related Protein (PTHrP) produced by Type II cells. The Type II cells secrete Prostaglandin E_2_, stimulating the secretion of the neutral lipids, and the uptake of the neutral lipid by the Type II cells is regulated by leptin produced by the lipofibroblasts. Each of these steps is coordinately stretch-regulated to increase surfactant phospholipid synthesis by the Type II cell. The net result is surfactant phospholipid production integrated with the distension of the alveolar wall during breathing.

**Figure 2:**
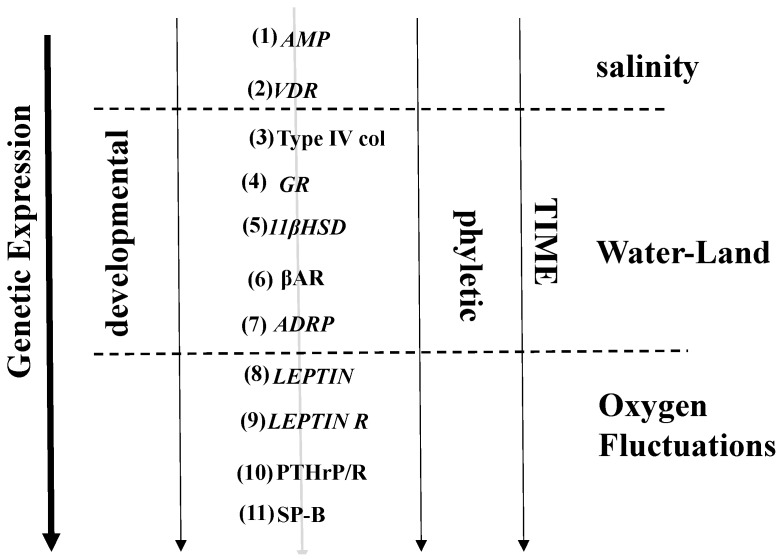
Pathways for the developmental and phyletic evolution of lipofibroblast-Type II cell interactions. Extrinsic selection pressures are shown in *italics*; intrinsic selection pressures are shown in bold. (1) AMPs = Antimicrobial Peptides; (2) VDR = Vitamin D Receptor; (3) Type IV col = Type IV collagen ; (4) GR = Glucocorticoid Receptor; (5) 11βHSD = 11beta Hydroxysteroid Dehydrogenase; (6) βAR = beta Adrenergic Receptor; (7) ADRP = Adipocyte Differentiation Related Protein; (8) Leptin = Leptin; (9) Leptin R = Leptin Receptor; (10) PTHrP = Parathyroid Hormone-related Protein; (11) SP-B = Surfactant Protein-B. These changes in genetic expression were sequentially brought about by such environmental factors as salinity, water-land transition and fluctuations in atmospheric oxygen tension over the last 500 million years.

The alveolar lipofibroblast (LIF) is a molecular Rosetta Stone [38], “translating” the developmental changes across vertebrate species into the evolution of the lung. The physiologic relevance of the LIF to alveolar growth, differentiation, homeostasis and repair has revealed such evolutionary homologies as (1) the peroxisome [39], which is thought to have evolved in response to the otherwise pathologic effects of Endoplasmic Reticulum stress in unicellular organisms; (2) Neutral Lipid Trafficking (Figure 1) [40], encompassing facilitated lipid uptake in defense against hyperoxia [41] mediated by Adipocyte Differentiation Related Protein (ADRP) storage [42], and release under the control of Prostaglandin E_2_ [43], referring all the way back to the biosynthesis and insertion of cholesterol into the eukaryotic cell membrane [8]; and (3) the secretion of the fat cell hormone leptin [44] to regulate surfactant production by the Alveolar Type II Cell (ATII), coming full circle from the antioxidant property of the LIF [41]. For orientation of these cellular-molecular evolutionary properties to the lung, the pathways for ontogeny, phylogeny and evolution of the LIF-ATII interactions are depicted in Figure 2.

LIFs in the alveolar wall of the rat lung were first described by Hitchcock *et al.* [45], and extensively documented in rodent [46,47,48] and more recently in human lung [49]. However, their functional relevance to the alveolus was not determined for two more decades, though their cytoprotective nature was suggested earlier by the comparative lung physiologic studies of Frank *et al.* [50], who showed the association between the LIFs and their putative role in antioxidant protection. These physiologic studies were paralleled by biochemical studies of triglyceride metabolism conducted by Mostello *et al.* [51].

The breakthrough in understanding the functional nature of these cells in lung alveolar physiology came with the co-culture of LIFs containing radiolabeled triglyceride and naive ATIIs, resulting in rapid passage of the tagged triglyceride from the LIF to the ATII, and their subsequent robust, enriched incorporation into surfactant phospholipid [52], termed Neutral Lipid Trafficking. Experimentally, it was observed that LIFs could readily take up triglyceride and store it in a stable form; furthermore, this process was under hormonal control by glucocorticoids, pointing to its regulated nature. Moreover, the presence of neutral lipid droplets in the LIFs protected them against oxidant injury [41], providing a function for these cells for the first time. It was subsequently determined that the uptake, storage and transit of the neutral lipids was actively mediated by ADRP, one of the proteins that mediate the trafficking and storage of neutral lipids throughout the body [53].

As mentioned above, during the course of these studies, it was empirically discovered that ATIIs could not absorb triglycerides (TGs) [52], whereas LIFs could not release them. That led to the discovery that Prostaglandin E_2_ (PGE_2_), secreted by ATIIs [43], specifically causes PGE_2_ receptor-mediated release of TGs by LIFs, and leptin produced by the LIFs facilitates the uptake of TGs by binding to its cell surface receptors on ATIIs [44].

The climax of these coordinated cell-cell interactions mediating and facilitating the production of lung surfactant was the discovery that Neutral Lipid Trafficking is stretch-regulated, providing key insights into both the cellular-molecular basis for the mechanism of alveolar ventilation-perfusion matching [54], and to the evolutionary history of the lung [54]. Initially, it was discovered that PTHrP is necessary for the formation of alveoli during lung morphogenesis [27], and that PTHrP secreted by the ATIIs stimulates LIF development, including TG uptake [55] and leptin secretion [44], providing evidence for the earliest epithelial signal to the mesenchyme during the course of the cell-cell interactions that mediate alveolar development. That, combined with the observation that PTHrP mRNA expression by ATIIs is stretch-regulated [56] led to the broader physiologic insight that the PTHrP Receptor (PTHrPR), leptin and the leptin receptor are all stretch-regulated signals, coordinating the physical distension of the alveolar wall with the “on-demand” up-regulation of surfactant production [40], promoting lung development *in utero* [57], and preventing alveolar atelectasis during air breathing [54]. The elucidation of this cellular-molecular mechanism for ventilation-perfusion matching is the first such evidence for the cellular-molecular basis for a physiologic property ever to be determined.

The ancestral relationship between the effect of stretch on PTHrP expression and microgravity was subsequently shown empirically. ATIIs were subjected to 0 × *g* conditions using a Rotating Wall Vessel Bioreactor [30]. The amount of PTHrP mRNA decreased over the first 8–12 h of microgravitational exposure, reaching a new stable baseline; when the cells were returned to unit gravity, the amount of PTHrP mRNA returned to its pre-microgravity exposure level. The deep significance of this relationship between alveolar regulation, mechanotransduction and lung development was revealed by study of the effect of microgravity on yeast [58]. Exposure to 0 × *g* caused loss of polarity and budding. The former is a reflection of the inability to mediate calcium flux [59], and the latter reflects the inability to reproduce [60]. These ancient physiologic properties refer all the way back to the unicellular state, when cholesterol facilitated eukaryotic evolution from prokaryotes, promoting metabolism, respiration and locomotion, the basic characteristics of vertebrate evolution [61].

These insights led to the realization that the endodermal and mesodermal components of the alveolar wall evolved over evolutionary time to generate their structural-functional properties through cell-cell interactions [8,36,62]. In support of that process, the PTHrPR duplicated during the vertebrate water-land transition some 300 mya (Million years ago) [25], amplifying the PTHrP signaling pathway in the lung, skin and bone. The causal nature of this interrelationship is evidenced by the deletion of PTHrP in developing mice, causing failure of lung, skin and bone development [27]. PTHrP is also expressed in the developing swim bladder along with many other genes expressed in lung [33], establishing the functional homology between these organs. Moreover, the gas gland epithelial cells that line the swim bladder secrete cholesterol, the most primitive lung surfactant, preventing the walls of the bladder from sticking to one another. That functional homology relates ancestrally all the way back to the advent of cholesterol in the cell membranes of evolving unicellular eukaryotes from prokaryotes, thinning the phospholipid bilayer, facilitating oxygenation, metabolism and locomotion, the fundamental properties of vertebrate physiologic evolution [61]. Cholesterol was subsequently coopted for the formation of lipid rafts, the structural site for cell-surface receptors, and much later were the substrate for steroid hormones and vitamin D, key elements of the endocrine system.

## 5. Physical Stress and Heterochrony—The Role of Gravity

Gravitational force is the oldest, constant, unidirectional force on Earth. As such, it affects biologic systems through mechanotransduction to affect cellular physiology. The fundamental nature of this effector is reflected by experiments in which yeast were exposed to microgravity, showing phenotypic effects on polarity and budding [58]. The former effect is a reflection of the role of gravity in calcium flux [59], the latter reflecting the effect of gravity on reproduction [60]. Experiments in our laboratory have similarly shown effects of microgravity on PTHrP expression in lung and bone cells *in vitro* [29], which were corroborated by assaying for PTHrP mRNA in the bones of rats flown in deep space for two weeks on NASA STS-58 [30]. The mRNA levels were significantly lower in the weight-bearing bones (tibia, femur) than in the non-weight bearing skull bones [30], consistent with the theory of mechanical effects on bone remodeling.

Affecting such fundamental adaptive mechanotransductive mechanisms has produced heterochronic changes over the history of organisms.

## 6. Physiologic Stress—The Role of Hypoxia

Oxygen has profoundly affected the evolution of terrestrial organisms, fueling their metabolism, causing increased growth [62] and differentiation [8]. This effect is most apparent over the course of the last 500 million years, during the Phanerozoic eon, oxygen rising and falling between 15% and 35% [63]. The increases have driven the growth of large insects and animals [64], whereas the subsequent decreases have profoundly affected visceral evolution since hypoxia is the most potent physiologic stressor. The residual of those insults is seen in the concerted evolution of the neuroendocrine [65], endocrine [66] and respiratory systems [8] of land vertebrates, hypothetically generating homeothermy/endothermy [10].

As added evidence for the interrelationship between physiologic stress and the co-evolution of the neuroendocrine and respiratory systems, lung surfactant has evolved to optimize surface tension reducing activity during the transition from poikilotherms to endotherms [66]. Initially, the stress of periodic hypoxia during land vertebrate evolution stimulated the hypothalamic-pituitary adrenal axis, increasing catecholamine secretion from the adrenal medulla [67]. Catecholamines stimulate surfactant secretion by the alveoli, making the alveoli more distensible, transiently relieving the hypoxic constraint on the evolving lung. Over time, the increased distension of the alveoli stimulates PTHrP secretion from the ATIIs, promoting alveolarization [27], providing a long-term solution for adaptation to air breathing [68]. In tandem, catecholamines stimulate fatty acid secretion from peripheral fat stores, increasing metabolism and body heat. The phospholipid composition of surfactant in the alveoli of land vertebrates has evolved through progressive increases in the percentage of dipalmitoylphosphatidylcholine (DPPC), which is 300% more bioactive at 37 °C than it is at 25 °C due to its increased phase transition temperature [66], offering a mechanistic explanation for the positive selection for DPPC [69]. In support of the causal effect of the ambient atmospheric temperature on lung surfactant phospholipid composition, Lau and Keogh [70] had shown such an interrelationship experimentally in MAP turtles. Moreover, hibernation, in association with decreased catecholamine production, demonstrates the opposite effect on the phosphatidylcholine content of lung surfactant [71], lending credence to the environmental effect on lung surfactant composition and surface tension reducing activity.

And since all of these properties are the net result of changes in cell-cell signaling mechanisms for structure and function, they can be characterized as heterochronies.

## 7. Chronic Lung Disease as “Reverse” Heterochrony

Many chronic diseases such as emphysema and Bronchopulmonary Dysplasia are characterized by structural simplification [72], atavistically reverting back to an earlier stage in their ontogeny and phylogeny. In the lung, this relationship has been well delineated by the recognition of the transdifferentiation of mesodermal fibroblasts from an adipocyte-like lipofibroblast to a myofibroblast, reversing their direction both developmentally [73] and phylogenetically [62]. The cause of this loss of differentiation is due to the breakdown in communication between the epithelial and mesenchymal components of the alveolar wall that fostered the growth and differentiation of the alveoli during development [7].

Similarly, modification of these cell-cell communications was responsible for the evolution of the lung from the swim bladder [10] phylogenetically, and ultimately in evolutionary adaptation to air breathing [12]. Thus, the heterochronic principle can be seen during the course of the reorientation of the cell-cell signaling mechanisms in the tissues that evolved to adapt to a novel environment [9,10]. As evidence of the causal nature of this mechanism, Peroxisome Proliferator Activated Receptor gamma (PPARγ) agonists [74] can prevent this loss of differentiated structure and function because it acts on the pathway that originally evolved to protect the lung against oxidant injury [41], referring all the way back in vertebrate phylogeny to a more general adaptation to oxidant injury by mesodermal cells [75].

PPARγ regulates peroxisome formation [76], and as such refers to the stage in eukaryote evolution when rising levels of oxygen caused Endoplasmic Reticulum Stress in unicellular organisms, resulting in calcium leaking into the cytoplasm, threatening to congeal nucleotides, proteins and lipids alike [77]. The evolutionary epistatic balancing mechanism was the Peroxisome [39], which utilizes lipids to buffer calcium dyshomeostasis. Therefore, the antecedents of the heterochronic redistribution of genetic expression can be seen in this pathobiologic model of loss and gain of homeostasis as the essence of evolution.

## 8. Goodpasture’s Syndrome as Waterproofing

Goodpasture’s Syndrome exhibits a similar evolutionary etiology. The disease state is due to the formation of autoantibodies against an isoform of Type IV collagen, namely Alpha 3(IV)NC1. It first appears phylogenetically in fish, and is omnipresent in amphibians, reptiles, mammals and birds. Evolutionarily, it is more hydrophobic than other Type IV collagen isoforms, preventing water loss across lung and kidney epithelial barriers in terrestrial vertebrates. Its appearance in land animals was probably due to this adaptive property.

Bearing in mind that the extracellular matrix is generated by cell-cell interactions, this isoform of Type IV collagen would have been the result of a heterochronic process.

## 9. Conclusions

The perspective expressed in this paper is that heterochrony is not a random mutational event, but instead is like Jacob’s “tinkering” mechanism [78], reallocating biologic properties for novel uses by “rewiring” cell-cell signaling mechanisms as the source of novel phenotypic change. Mutations can occur within a specific biologic context, resulting in change consistent with the prevailing physical constraint when “deciphered” by the biologic cell-cell signaling. This has been true right from the inception of life itself, the micelle providing a protected environment for the evolution of catalysis, negentropy, chemiosmosis and homeostasis in order to cope with the vicissitudes of perpetual environmental change [79]. The key to understanding this process is in focusing on the communication between the organism and its environment, internalizing and compartmentalizing toxic substances (oxygen, ions, heavy metals) that would otherwise have destroyed it, forming physiologic systems in the process [8]. The subsequent formation of multicellular organisms was predicated on cell-cell communication for further adaptation, but always returning to the unicellular state, perhaps because it is the unicellular state that is the primary level of selection [10].

By seeing the process of evolution as communication, novel insights are gained that would otherwise remain tautologies and dogma [80]. Kuhn [14] defined a paradigm shift as a change in the language. The shift from heterochrony as a descriptive change in timing to alterations in developmental-homeostatic mechanisms changes the language. This is analogous to the paradigm shift in our understanding of gravity from Newton’s Law of Gravity that described the process to Einstein’s explanation of gravity as the distortion of space-time.
